# APY0201 Represses Tumor Growth through Inhibiting Autophagy in Gastric Cancer Cells

**DOI:** 10.1155/2022/7104592

**Published:** 2022-06-24

**Authors:** Huan Li, Xinghan Jin, Shiwei Zhang, Bo Li, Leli Zeng, Yulong He, Changhua Zhang

**Affiliations:** Guangdong Provincial Key Laboratory of Digestive Cancer Research, Digestive Diseases Center, The Seventh Affiliated Hospital of Sun Yat-Sen University, Shenzhen, Guangdong 518107, China

## Abstract

Gastric cancer (GC) is one of the most common cancers globally. There are currently few effective chemotherapeutic drugs available for GC patients. The inhibitors of phosphatidylinositol kinase containing an FYVE finger structure (PIKfyve) have shown significant anticancer effects in several types of cancers, but their effectiveness in GC remains unknown. In this study, we investigate the effect of APY0201, an inhibitor of PIKfyve, on GC tumor growth and its mechanism of action. It was found that APY0201 inhibited GC cell proliferation in *in vitro* GC cell model, organoid model, and *in vivo* xenograft tumor model. Through analyzing cell autophagy, we found that APY0201 might block autophagic flux by impairing lysosome degradation function of GC cells, inducing the accumulation of autophagosomes, thus causing the inhibition of GC cell proliferation. We also found that APY0201 induced G1/S phase arrest in GC cells. Importantly, APY0201 was also effective in inducing repression of autophagy and cell cycle arrest in the mouse tumor xenograft. Our results suggest that APY0201 could be a new promising therapeutic option for GC.

## 1. Introduction

Gastric cancer (GC) is one of the most common cancers, and its morbidity and mortality rates rank the fifth and fourth, respectively, globally [1]. Chemotherapy is the main treatment for unresectable advanced GC [2]. However, primary or acquired chemoresistance causes poor prognosis of GC patients [3]. At present, there is a lack of effective chemotherapeutic drugs against GC; thus, developing new chemotherapeutic drugs for the treatment of GC is critical and urgent.

It is known that the phosphatidylinositol kinase containing an FYVE finger structure (PIKfyve) is closely associated with lysosomal function and autophagy [4, 5]. Several studies have demonstrated that PIKfyve inhibitors exert an antitumor effect in some malignant tumors, and this effect is closely related to autophagy [6–8]. Hayakawa et al. first identified APY0201 as a small-molecule inhibitor of PIKfyve [9]. De Campos et al. reported that APY0201 showed a stronger antitumor effect than other members of the same type of PIKfyve inhibitors (Apilimod, YM201636) in multiple myeloma [10]. However, the effects of PIKfyve inhibitors, including APY0201, in GC have not been reported.

Autophagy is a highly conserved intracellular catabolic process in evolution. Autophagy can convert damaged organelles, proteins, and other substances into energy metabolites to maintain cell homeostasis [11, 12]. Autophagy dysfunction is closely related to cancer, immune dysfunction, and other diseases [13]. Several investigations have found that inhibiting autophagy can repress GC cells from proliferating [14–16]. In addition, the cell cycle is a highly regulated process responsible for cell growth, genetic material replication, and cell division [17]. The complexes formed by cyclins and cyclin-dependent kinases (CDKs) play critical roles in cell cycle regulation [18]. The genetic mutations of cancer cells render them from exiting the cell cycle, allowing cell division to continue [19]. As such, cell cycle has been exploited as a therapeutic target in anticancer treatments to prevent cancer cells from dividing in cancer progression [20].

In this study, we showed that APY0201 induced significant inhibition of GC cell proliferation both *in vivo* and *in vitro*. APY0201 blocked the autophagic flux of GC cells by damaging the lysosomal degradation function, causing accumulation of autophagosomes, thereby inhibiting the proliferation of GC cells. Moreover, APY0201 induced G1/S phase arrest in GC cells. These findings suggest that APY0201 has the potential to be a new GC therapeutic agent.

## 2. Materials and Methods

### 2.1. Reagents and Antibodies

APY0201, bafilomycin A1 (BafA1), and rapamycin (Rapa) were obtained from MedChemExpress (MCE, Shanghai, China) and were dissolved in DMSO (Sigma, USA) for *in vitro* experiments. APY0201 was dissolved in PEG300 (MCE, Shanghai, China) for animal research *in vivo*. Anti-LC3A/B (#12741S), CTSD (#2284S), CTSB (#311718T), CDK2 (#2546T), CDK4 (#12790T), cyclin E1 (#4129T), p21 (#2947T), p27 (#3686T), and ATG5 (#12994T) were obtained from Cell Signaling Technology (Danvers, USA). Anti-p62 (#PM045) was obtained from MBL (Tokyo, Japan). Anti-CDK6 (#ab124821) and Anti-Ki67 (#ab15580) were obtained from Abcam (Cambridge, UK). Anti-cyclin D1 (#ab143498) and GAPDH (#60004-1-Ig) were obtained from Proteintech (Wuhan, China). Anti-tubulin (#AF0001) was obtained from Beyotime (Shanghai, China).

### 2.2. Cell Culture

Human GC cell lines including AGS, SGC7901, MKN28, BGC823, and SNU719 were bought from the Cell Bank of Chinese Academy of Sciences (Shanghai, China). All cells were grown in RPMI1640 media (Gibco, USA) with 10% fetal bovine serum (FBS, Gibco, USA) and 1% penicillin/streptomycin (Gibco, USA) at 37°C, 5% CO_2_, and 95% humidity in an incubator.

### 2.3. Cell Viability Analysis

Cell viability was determined by the Cell Counting Kit-8 (CCK-8, Meilun Biotech, China) assay. AGS, SGC7901, MKN28, BGC823, and SNU719 cells were plated on 96-well plates (3 × 10^3^ cells/well). The cells were treated with various concentrations of APY0201 (0 *μ*M, 0.01 *μ*M, 0.1 *μ*M, 0.5 *μ*M, 1 *μ*M, 5 *μ*M, 10 *μ*M) for 48 h. Then 10 *μ*l CCK-8 reagent and 90 *μ*l medium were added to each well, followed by a 2 h incubation period in the dark. A Synergy H1 microplate reader (BioTek, USA) was used to measure light absorbance at 450 nm wavelength.

### 2.4. Colony Formation Assay

AGS and SGC7901 cells were seeded on 6-well plates (600 cells/well). Cells were incubated with APY0201 at various concentrations (0 *μ*M, 0.1 *μ*M, 1 *μ*M). Cells were fixed with 4% paraformaldehyde (Biosharp, China) after 14 days of continuous culture and stained for 30 minutes with 0.1% crystal violet. Photographs and counts of clones were taken.

### 2.5. EdU Cell Proliferation Assay

Cell proliferation was evaluated using an EdU assay kit (Beyotime, China). AGS and SGC7901 cells were seeded into 96-well plates (3 × 10^3^ cells/well) and treated for 48 h with APY0201 at various concentrations (0 *μ*M, 0.1 *μ*M, 1 *μ*M). After addition of EdU reagent for 2 h, the cells were fixed, cleaned, and subjected to EdU reaction and nuclear staining. An inverted fluorescence microscope (Leica, Germany) was used to view the EdU-stained samples.

### 2.6. Human Tissues and Organoids

Human gastric cancer tissues were collected from five GC patients who underwent surgery in the Seventh Affiliated Hospital of Sun Yat-sen University. All participated patients signed the informed consent forms. The ethics committee of Sun Yat-sen University's Seventh Affiliated Hospital authorized this study (KY-2020-042-01). Five organoids (named GC1, GC2, GC3, GC4, and GC5) from GC patients were selected for the experiment. The establishment and culture methods of organoids were in accordance with our previous research [21].

### 2.7. Morphological Records and Cell Viability Analysis of Organoids

Five GC organoids (GC1, GC2, GC3, GC4, GC5) were seeded in 96-well plates (3 × 10^3^ cells/well) containing Matrigel and were cultured for three days. They were given APY0201 at various concentrations (0 *μ*M, 1 *μ*M, 2.5 *μ*M, 5 *μ*M). Three days later, the organoids' morphology was examined and photographed with an optical microscope, and the cell viability was assessed by CCK8 assay as described above in GC cells.

### 2.8. Cell Cycle Analysis

The cell cycle was studied by a cell cycle staining kit (Multisciences, China). AGS and SGC7901 cells were plated on 6-well plates (2 × 10^5^ cells/well). DMSO and APY0201 (1 *μ*M) were applied to the cells. After 24 h, DNA staining solution and osmotic solution were added to the cells, followed by 30 minutes of culture. Finally, cells were subjected to the flow cytometer (Beckman, USA) for cell cycle analysis and the results were analyzed with FlowJo software (Tree Star, USA).

### 2.9. Western Blot Analysis

Cells or nude mouse tumor samples were collected, followed by the addition of RIPA protein lysis solution (Beyotime, China). Protein concentration was determined by BCA protein analysis kit (Beyotime, China). Then proteins were separated by 10–15% SDS-PAGE and were transferred to PVDF membranes (Millipore, USA) subsequently. The membranes were sealed in 5% BSA for 1 h then incubated with primary antibodies at 4°C overnight. The free antibodies were removed by TBST and the HRP-linked secondary antibodies (Beyotime, China) were applied to the membranes for 1 h. The ChemiDocTM MP system (Bio-Rad, USA) was used to photograph the membranes utilizing an enhanced chemiluminescence (ECL) kit (Beyotime, China).

### 2.10. Autophagic Flux Analysis

AGS or SGC7901 cells were seeded onto 6-well plates (2 × 10^5^ cells/well). Cells were infected with lentiviral vectors expressing the StubRFP-SensGFP-LC3 gene (GeneChem, Shanghai, China). Three days later, puromycin (2 g/ml) was added to the medium, and the successfully infected cells were obtained after 7 days of culture. Then cells were seeded into confocal dishes (1 × 10^4^ cells/well). AGS and SGC7901 cells were treated with DMSO, APY0201 (0.01 *μ*M, 0.1 *μ*M, 1 *μ*M), and Rapa (2 *μ*M), or BafA1 (10 nM) for 24 h and fixed with 4% paraformaldehyde. Autophagic flux was then observed using a confocal Zeiss LSM900 microscope (Carl Zeiss, Germany).

### 2.11. Short Hairpin RNA (shRNA) Transfection

Lentivirus vector expressing shATG5 sequence (GCCAAGTAAGTTATTTGAC) were purchased from FulenGen (Guangzhou, China). AGS or SGC7901 cells were inoculated into six-well plates (2 × 10^5^ cells/well). Cells were infected with lentiviral vectors expressing the shNC and shATG5 gene. Three days later, puromycin (2 g/ml) was added to the medium, and the successfully infected cells were obtained after 7 days of culture. The AGS or SGC7901 cells were inoculated into a 6 cm dish or 96-well plate, treated with DMSO and APY0201 (1 *μ*M), respectively, and then subjected to western blot and CCK8 assays.

### 2.12. *In Vivo* Tumor Xenograft Model

Four-week-old male nude BALB/c mice were obtained from Beijing Weitong Lihua Experimental Animal Science and Technology Co., Ltd. (China). The ethical committee of Shenzhen Top Biotechnology Co., Ltd.'s SPF Experimental Animal Center authorized this study (TOP-IACUC-2021-0105). A total of 8 × 10^6^ SGC7901 cells were delivered into the right back of each nude mouse by subcutaneous injection. Nude mice were randomly separated into control and APY0201 treatment groups after tumor volumes reached about 80 mm^3^ (8 mice per group). The control group and the APY0201 treatment group were intraperitoneally injected with excipient and APY0201 (30 mg/kg, dissolved in 50% PEG300 + 50% saline), respectively, once per day for 14 days. The length (L) and width (W) of tumors were measured every 2 days. The tumor volume (V) = 0.5 × L × W^2^. The nude mice were weighed every 2 days. After the experiment, the nude mice were sacrificed and the tumors were collected and photographed. Parts of the tumors were fixed with formalin for the immunohistochemical experiment. The remainder of the tumors were transferred to liquid nitrogen for western blotting.

### 2.13. Immunohistochemical Analysis

Paraffin embedded subcutaneous tumor tissues of nude mice were made into sections. The sections were dewaxed with xylene and rehydrated with ethanol, and then the antigen was retrieved by 1x Tris-EDTA buffer in a pressure cooker at 95°C for 10 min. Following a 20-minute incubation in a 3% hydrogen peroxide solution, the slices were blocked with 5% goat serum for 30 minutes before being treated with the primary antibodies overnight. This was followed by a 1 h incubation in which they were exposed to the secondary antibody. DAB solution was used for dyeing the sections. Light microscopy was used to examine and photograph the sections after they had been counterstained with hematoxylin.

### 2.14. Statistical Analysis

GraphPad Prism 9.0 (GraphPad Software, USA) and SPSS 26.0 (IBM Corp., USA) were used to conduct statistical analysis. Data were presented as mean ± SD. The data were analyzed using Student's *t*-test and one-way ANOVA. *P* < 0.05 was deemed to be a statistically significant difference.

## 3. Results

### 3.1. APY0201 Inhibits Proliferation of GC Cells

**Figure 1:**
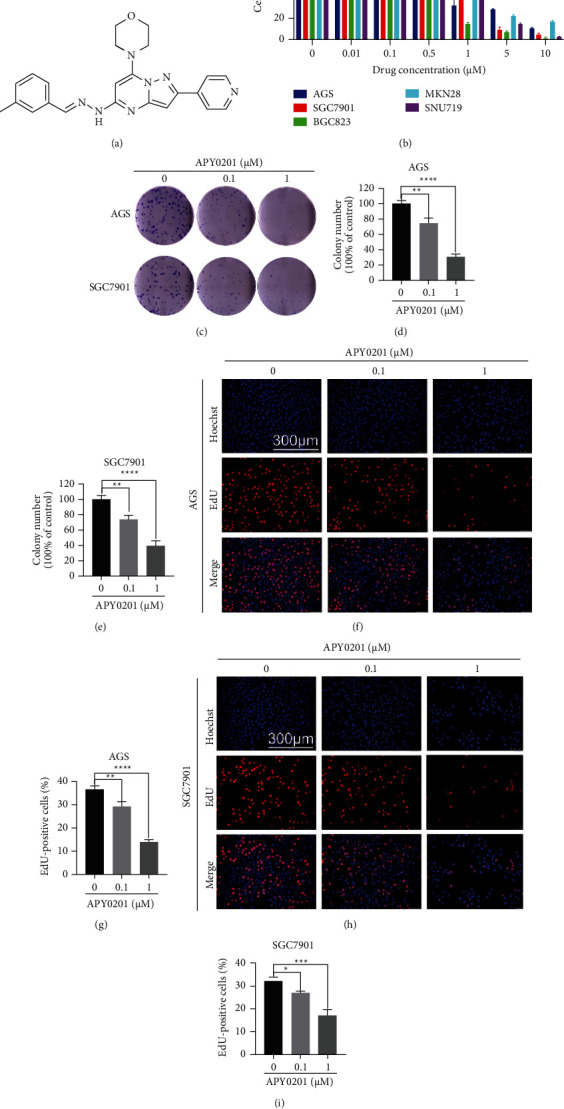
APY0201 inhibits proliferation of GC cells. (a) Molecular structure of APY0201. (b) AGS, SGC7901, BGC823, MKN28, and SNU719 cells were treated with varied concentrations of APY0201 for 48 h and cell viability was determined. (c) Colony formation in AGS and SGC7901 cells treated with various concentrations of APY0201 for 14 days. (d)-(e) Statistical analysis of the numbers of colonies in part c. (f), (h) The EdU proliferation test was used to investigate the effects of varied concentrations of APY0201 on AGS and SGC7901 cells for 48 h. The scale bar is 300 *μ*m. (g), (i) Proportions of EdU-positive cells in AGS and SGC7901 cells. ^*∗*^*P* < 0.05, ^*∗∗*^*P* < 0.01, ^*∗∗∗*^*P* < 0.001, ^*∗∗∗∗*^*P* < 0.0001 compared with 0 *μ*M APY0201 group.

The molecular structure of APY0201 (MW 413.48) used in this study is shown in Figure 1(a). The effect of APY0201 on GC cell proliferation was first confirmed by using the CCK8 assay, which revealed that APY0201 decreased the viability of AGS, SGC7901, BGC823, MKN28, and SNU719 cells in a concentration-dependent manner (Figure 1(b)). Following that, we found that APY0201 reduced the number of colony formation of AGS and SGC7901 cells in a concentration-dependent manner (Figures 1(c)–1(e)). Finally, we found that APY0201 reduced the number of EdU-positive cells in AGS and SGC7901 cells in a concentration-dependent manner (Figures 1(f)–1(i)).

### 3.2. APY0201 Inhibits the Growth of GC Organoids

**Figure 2:**
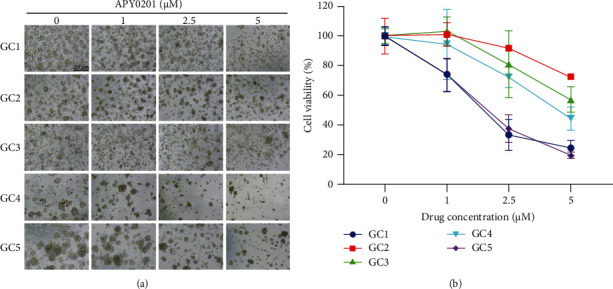
APY0201 inhibits the growth of GC organoids. GC organoids were treated with various concentrations of APY0201 for 3 days. (a) Light microscope photos of GC organoids. The scale bar is 300 *μ*m. (b) GC organoids were tested for cell viability by the CCK8 assay, and a broken line graph was created to illustrate this.

We tested the impact of APY0201 on GC cell proliferation by using a GC organoid model. We treated five GC organoids with different concentrations of APY0201. After the drug treatment, we first observed and photographed the organoids under an optical microscope and then carried out a CCK8 assay. As shown in Figure 2(a), APY0201 was found to reduce the size and number of all organoids in a concentration-dependent manner. There is a concentration-dependent inhibition of cell viability in all organoids by APY0201 (Figure 2(b)).

### 3.3. APY0201 Inhibits the Growth of GC Xenografts

**Figure 3:**
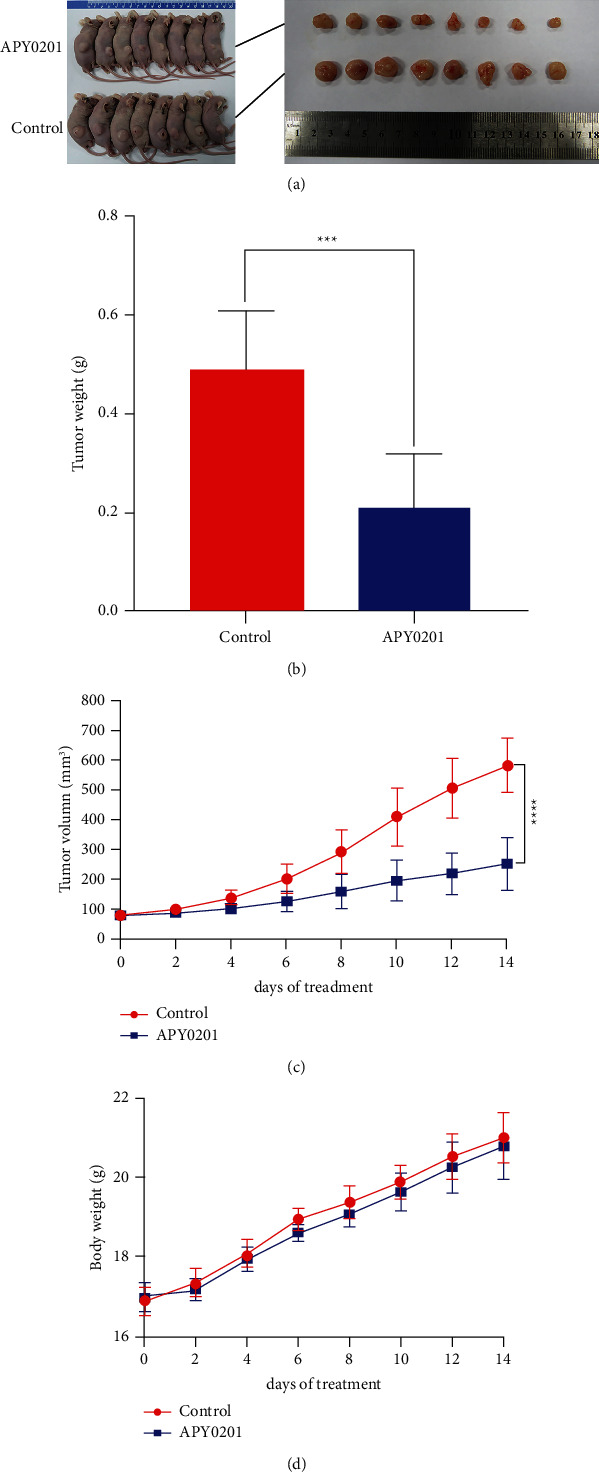
APY0201 inhibits the growth of GC xenografts. (a) Photographs of subcutaneous transplanted tumors and nude mice. (b) The average weight of transplanted tumors. (c) The volume of the transplanted tumor in each nude mouse was measured every 2 days, and a tumor growth curve during APY0201 treatment was plotted. (d) Mice were weighed every 2 days and a body weight change curve during APY0201 treatment was plotted.

To demonstrate the antitumor activity of APY0201 *in vivo*, we constructed a GC subcutaneous xenograft model in nude mice. The results demonstrated that the APY0201 group had lower volumes and weights of transplanted tumors than the control group (Figures 3(a) and 3(b)) and that their growth rates were slower than the control group (Figure 3(c)). Moreover, the body weight of the APY0201 treated mice was found to not differ significantly within the 14-day treatment period compared to the control groups (Figure 3(d)).

### 3.4. APY0201 Induces Autophagosome Accumulation

**Figure 4:**
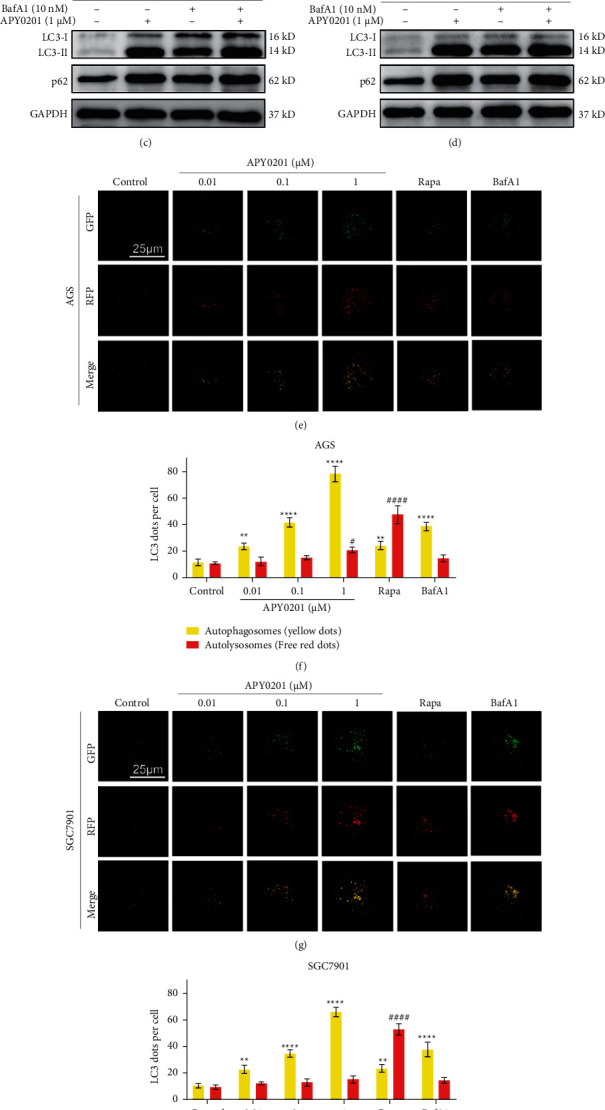
APY0201 induces autophagosome accumulation. (a)-(b) AGS and SGC7901 cells were treated with APY0201 at various concentrations for 24 h and the expressions of LC3 and p62 were determined by western blotting. (c)-(d) AGS and SGC7901 cells were treated with DMSO and APY0201 with or without BafA1 for 24 h and the expressions of LC3 and p62 were determined by western blotting. (e), (g) AGS and SGC7901 cells stably expressing StubRFP-SensGFP-LC3 were seeded onto confocal culture dishes and treated with different drugs for 24 h. Confocal microscopy revealed the development of LC3 dots in AGS and SGC7901 cells (*λ*_ex/green_ = 488 nm, *λ*_em/green_ = 500–530 nm; *λ*_ex/red_ = 545 nm, *λ*_em/red_ = 590–630 nm). The scale bar is 25 *μ*m. (f), (h) Histograms were based on the numbers of yellow and red dots in different drug groups shown in parts (e) and (g). ^*∗*^*P* < 0.05, ^*∗∗*^*P* < 0.01, ^*∗∗∗*^*P* < 0.001, ^*∗∗∗∗*^*P* < 0.0001 were compared with autophagosomes in the control group; ^#^*P* < 0.05, ^##^*P* < 0.01, ^###^*P* < 0.001, ^####^*P* < 0.0001 were compared with autolysosomes in the control group.

As shown in Figures 4(a) and 4(b), APY0201 significantly increased LC3-II and p62 expression levels in GC cells in a concentration-dependent manner. The expression level of LC3-II represents the level of autophagosomes in cells [22]. The increase in autophagosomes in cells can be attributed to two factors: an increase or an interruption of autophagic flux [23]. p62 binds to LC3-II on the autophagosome membrane to form a complex, which is then degraded in lysosome [24]. Therefore, p62 is negatively correlated with autophagic flux. These results demonstrated that APY0201 caused autophagosome accumulation most likely due to the blockage of autophagic flux in GC cells. Using BafA1, an inhibitor of late autophagy, which damages lysosome function by inhibiting V-ATPase, thereby blocking autophagic flux [25], we found that APY0201 in combination with BafA1 did not raise LC3-II and p62 expression in AGS and SGC7901 cells when compared to APY0201 alone, as demonstrated by western blot analysis (Figures 4(c) and 4(d)).

To further verify the influence of APY0201 on autophagic flux, AGS or SGC7901 cells were infected with tandem StubRFP-SensGFP-LC3 lentivirus, and the formation of LC3 dots can be observed under a confocal microscope. GFP is quenched after entering the lysosome owing to the pH value within the lysosome which is less than 5, whereas RFP is not affected by pH. Thus, the yellow dots formed by GFP and RFP together represent autophagosomes, whereas the red dots represent autolysosomes. In comparison to the control group, APY0201 increased the number of autophagosomes in AGS and SGC7901 cells in a concentration-dependent manner, and the number of autophagosomes was greater than that of autolysosomes after treatment of APY0201 (Figures 4(e)–4(h)). Similar to the autophagy inhibitor BafA1, the APY0201 can also cause a large increase in numbers of autophagosomes (Figures 4(e)–4(h)). However, the effect of APY0201 was opposite to that of autophagy inducer Rapa (Figures 4(e)–4(h)). In conclusion, APY0201 can block the autophagic flux of GC cells, thereby causing autophagosome accumulation.

### 3.5. APY0201 Impairs the Degradation Function of Lysosomes by Inhibiting the Activity of Cathepsin in GC Cells

**Figure 5:**
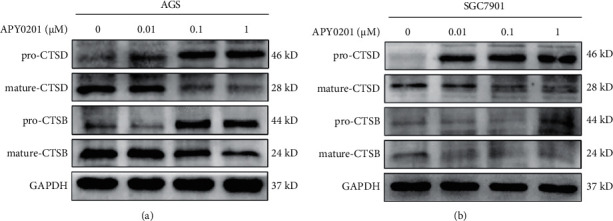
APY0201 impairs the degradation function of lysosomes by inhibiting the activity of cathepsin in GC cells. (a)-(b) AGS and SGC7901 cells were treated with APY0201 at various concentrations for 24 h and the expressions of CTSD and CTSB were detected by western blotting.

Autophagy dysfunction is usually caused by defects in lysosomal activity [26]. Cathepsins are a group of heterogeneous proteases located in lysosomes that are mainly responsible for protein hydrolysis in acidic environments and are closely related to the degradation function of lysosomes [27, 28]. Therefore, we speculated that APY0201 would impair lysosomal degradation function by inhibiting cathepsin activity in GC cells. Studies have shown that a lack of cathepsin *D* (CTSD) and cathepsin *B* (CTSB) can lead to serious damage to the lysosomal autophagy [29, 30]. We used western blotting to explore the expression of CTSD and CTSB in AGS and SGC7901 cells treated with different concentrations of APY0201. The results indicated that APY0201 increased the expression of pro-CTSD and pro-CTSB proteins in AGS and SGC7901 cells, while it decreased the expression of mature CTSD and CTSB proteins (Figures 5(a) and 5(b)). The degradation function of lysosomes was harmed by a large rise in inactive procathepsins and a corresponding decrease in mature cathepsins. These findings demonstrate that APY0201 impairs the degradation function of lysosomes by inhibiting cathepsin activity in GC cells.

### 3.6. APY0201-Induced Autophagosome Accumulation Inhibits Proliferation of GC Cells

**Figure 6:**
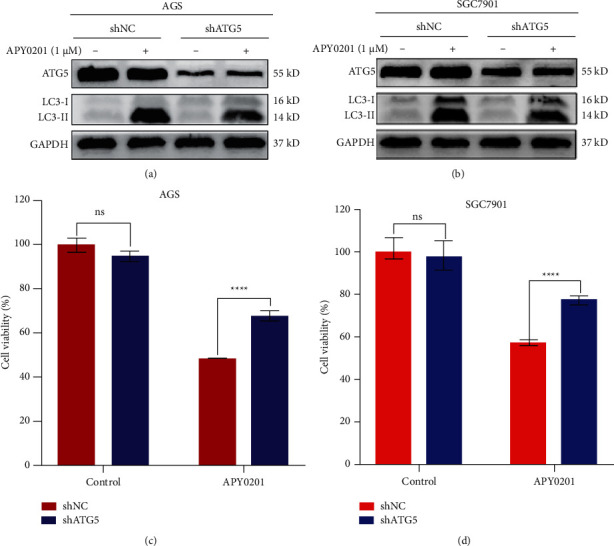
APY0201-induced autophagosome accumulation inhibits proliferation of GC cells. AGS and SGC7901 cells were infected with lentiviruses carrying shNC and shATG5 genes. Subsequent experiments were carried out with AGS and SGC7901 cells stably expressing shNC and shATG5. (a, b) After 24 h of treatment with DMSO and APY0201, AGS and SGC7901 cells were examined by western blotting for ATG5 and LC3 expression. (c), (d) DMSO and APY0201 were applied to AGS and SGC7901 cells for 48 h and their viability was determined by CCK8 assay. Histograms were then drawn. ^*∗∗∗∗*^*P* < 0.0001; ns, no statistically significant difference compared with the shNC group.

APY0201 inhibited the proliferation of GC cells and increased the number of autophagosomes in GC cells in a concentration-dependent manner (Figures 1 and 4). Damaged organelles and macromolecular proteins in autophagosomes can be transformed into energy metabolites such as nucleotides and amino acids after degradation in lysosomes to maintain cell homeostasis [11, 12]. Large numbers of undegraded autophagosomes may have toxic effects on GC cells. Therefore, we speculated that the accumulation of autophagosomes caused by APY0201 could inhibit the proliferation of GC cells. Autophagy-related gene 5 (ATG5) is essential in the formation of autophagosomes, and knockdown or knockout of ATG5 leads to partial or complete inhibition of autophagy [31, 32]. To test the link between autophagosome accumulation and GC cell proliferation, AGS and SGC7901 cells with stable expression of shNC and shATG5 were constructed and treated with APY0201. Western blotting analysis revealed that the shATG5 group had significantly lower levels of LC3-II expression than the shNC group following APY0201 treatment (Figures 6(a) and 6(b)), indicating that the autophagosome accumulation induced by APY0201 was significantly reduced following ATG5 knockdown. Cell viability in the shATG5 group was higher than the shNC group following APY0201 treatment, according to CCK8 assay results (Figures 6(c) and 6(d)). These findings indicated that ATG5 knockdown reduced the autophagosome accumulation generated by APY0201 and partially reversed the inhibitory effect of APY0201 on GC cell proliferation. These results suggested that autophagosome accumulation induced by APY0201 inhibits the proliferation of GC cells.

### 3.7. APY0201 Induces G1/S Phase Arrest of GC Cells

**Figure 7:**
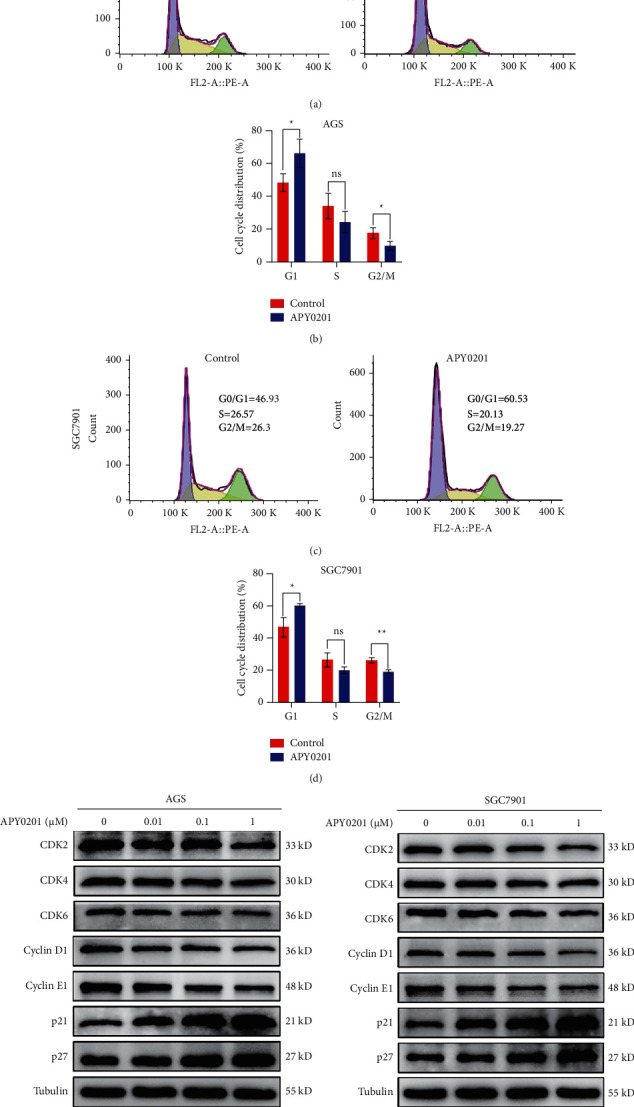
APY0201 induces G1/S phase arrest of GC cells. (a), (c) After 24 h of DMSO and APY0201 treatment, the cell cycle of AGS and SGC7901 cells was examined by flow cytometry. (b), (d) Percentage distribution of cell cycle phases in the control group and APY0201 group. ^*∗*^*P* < 0.05, ^*∗∗*^*P* < 0.01; ns, no statistically significant difference compared with control group. (e), (f) AGS and SGC7901 cells were treated with APY0201 at various concentrations for 24 h. The expression levels of CDK2, CDK4, CDK6, cyclin D1, cyclin E1, p21, and p27 were analyzed by western blotting.

Previous studies have demonstrated that GC cell proliferation is tightly tied to cell cycle and that cell cycle arrest caused by small-molecule drugs can suppress GC cell proliferation [33–35]. In this study, the effects of APY0201 on the cell cycle of AGS and SGC7901 cells were detected by flow cytometry. For both cell types, the proportion of cells in G1 phase in the APY0201 group increased compared with the control group, whereas the proportion of cells in S phase and G2/M phase decreased (Figures 7(a)–7(d)). To validate the flow cytometry results, we extracted the protein lysis products from AGS or SGC7901 cells treated with various concentrations of APY0201 and then used western blotting to determine the expression levels of cell cycle regulatory proteins that play critical roles in the transition from G1 to S phase. The results showed that APY0201 reduced the expression of CDK2, CDK4, CDK6, cyclin D1, and cyclin E1 but increased the expression of p21 and p27 in AGS and SGC7901 cells in a concentration-dependent manner (Figures 7(e) and 7(f)).

### 3.8. APY0201 Inhibits the Proliferation of GC Transplanted Tumor Cells by Interrupting Autophagic Flux and Cell Cycle Arrest

**Figure 8:**
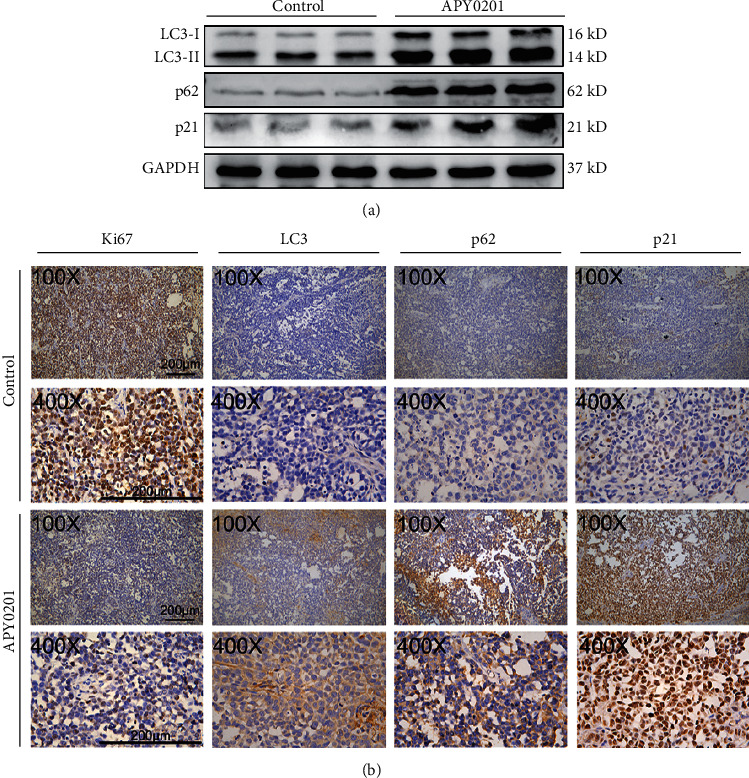
APY0201 inhibits the proliferation of GC transplanted tumor cells by interrupting autophagic flux and cell cycle arrest. (a) The expression of LC3, p62, and p21 in transplanted tumors was analyzed by western blotting. (b) The expression of Ki67, LC3, p62, and p21 in transplanted tumors was detected by immunohistochemistry. The scale bar is 200 *μ*m.

The protein lysis products of transplanted tumor tissues were examined by western blotting. The LC3-II, p62, and p21 expression levels in the APY0201 group were considerably greater than those in the control group (Figure 8(a)). Immunohistochemistry was performed on paraffin slices of the transplanted tumor tissues. The APY0201 group had remarkable higher expression levels of LC3, p62, and p21 than those in the control group, whereas the expression level of proliferation marker Ki67 in the APY0201 group was significantly lower than that in the control group (Figure 8(b)). Therefore, the results indicated that APY0201 could inhibit the GC tumor growth via interrupting autophagic flux and cell cycle arrest.

## 4. Discussion

GC is a deadly malignant tumor with a high fatality rate. The known risk factors for GC include family history of GC, diet, alcohol consumption, smoking, *Helicobacter pylori,* and EB virus infection, among which the most common risk factor is *Helicobacter pylori* infection [36]. *Helicobacter pylori* works through a variety of virulence factors, making it colonize the gastric mucosa, leading to gastritis or gastric ulcer, which greatly increases the risk of GC [37, 38]. The 5-year survival rate of patients with early GC is more than 90%, while that of patients with advanced GC is only about 10% [39, 40]. Endoscopic submucosal dissection (ESD) has been accepted as the standard surgical method for the treatment of early GC, and ESD as an effective treatment has been proved to be related to the good long-term prognosis of patients with early GC [41–43]. Most GC patients are diagnosed at advanced stage and have lost the opportunity for surgery. Nonsurgical treatment with chemotherapeutic drugs is particularly important for patients with advanced GC. However, owing to the existence of chemoresistance, the therapeutic effect of chemotherapeutic drugs in GC is limited, and only a small number of patients with advanced GC would benefit from chemotherapy [44, 45]. In recent years, many chemotherapeutic drugs for the treatment of GC have emerged; however, they have not shown satisfactory therapeutic effect. At present, a number of studies have proved that some small molecular compounds and plant extracts show strong antitumor effects in GC, which reflected their great potential as therapeutic drugs for GC [46–49]. In this research, we explored the antitumor effect and mechanism of APY0201 in GC as a potential new therapeutic strategy.

*In vitro* cell proliferation assays revealed that APY0201 inhibits GC cell proliferation. APY0201 inhibited the growth of transplanted tumors *in vivo* and had no significant effect on the body weight of the mice. APY0201 decreased the expression level of Ki67 in GC transplanted tumor tissue. These results show that APY0201 can suppress the cell proliferation in GC, indicating that it is a promising candidate medication for the treatment of GC.

Organoids are three-dimensional tissue structures formed by self-organizing stem cells extracted from healthy or diseased individuals *in vitro* [50]. They can be used to simulate key structural and functional characteristics of the corresponding organs *in vivo* [51]. Organoids have displayed great potential in clinical applications, especially in anticancer drug screening, and their emergence as a new *in vitro* model system has contributed the development of the basic and translational research in cancer therapy [52, 53]. Compared with traditional 2D culture, organoid culture has higher tumor heterogeneity and stability. Meanwhile, organoid culture is simple and feasible, and the cost of organoid culture is significantly lower than that of animal experimental research. Organoids can be used as a bridge between 2D culture and animal models to increase experimental credibility. In this study, APY0201 was shown to inhibit the growth of all GC organoids. As tumor organoids can show the same drug response as the corresponding tumors *in vivo* after exposure to therapeutic drugs, these data also demonstrate APY0201's potential clinical utility in the therapy of GC patients.

Autophagy is a mechanism that turns intracellular components and malfunctioning organelles to lysosomes for degradation and recycling [54]. In the early stage of tumor formation, autophagy, as a survival pathway and quality control mechanism, can prevent tumor formation and inhibit tumor progression. Once the tumor has been formed, especially when it reaches the advanced stage, autophagy functions as a dynamic degradation and circulation system that can provide the energy required for tumor cell survival and development [55, 56]. Autophagy inhibition may be an effective therapy option for advanced cancers [57]. In recent years, increasing numbers of studies have used autophagy inhibitors to different malignant tumors and shown that it may have strong antitumor effects [58–60]. In this study, we demonstrated that APY0201 blocked autophagic flux and caused autophagosome accumulation in GC cells. We demonstrated that APY0201 caused autophagic flux interruption in GC transplanted tumor cells. The lysosome, a complex signaling center which controls cell growth, division, and differentiation, is closely related to cancer, neurodegeneration, and other diseases. The lysosome is crucial for quality control and stress adaptation of cells, which participates the process of autophagy [61]. In this study, we demonstrated that APY0201 impairs lysosomal degradation by inhibiting lysosomal cathepsin activity. As such, APY0201 might impair lysosomal degradation by inhibiting cathepsin activity in GC cells, thereby blocking autophagic flux and leading to autophagosome accumulation.

Several studies have shown that autophagosome accumulation caused by drug blocking of autophagic flux leads to tumor cell death [62–64]. The accumulation of autophagosomes can lead to the accumulation of many damaged organelles and proteins, which may be the direct cause of autophagosome accumulation leading to cell death. To verify the relationship between the accumulation of autophagosomes and the cell proliferation in GC cells, we constructed GC cell lines with stable shATG5 expression and subjected them to western blot and CCK8 assays. The results showed that autophagosome accumulation induced by APY0201 inhibited the proliferation of GC cells.

Recently, cell cycle inhibitors have shown remarkable antitumor properties on several malignancies, indicating their potential in tumor therapy [65–67]. The advancement of four distinct phases of the cell cycle, which are regulated by cyclins and CDKs, is required for cell proliferation [68]. p21 and p27 are well-known CDK inhibitors (CKIs) that suppress the activity of cyclin-CDK complexes and thereby prevent the transition from G1 to S phase [69, 70]. Flow cytometry data revealed that APY0201 caused G1/S phase arrest in GC cells. Our subsequent western blot analysis revealed that APY0201 decreased the expression of CDK and cyclin-related proteins involved in the transformation of GC cells from G1 to S phase. Furthermore, APY0201 increased expression levels of CKI-related proteins that block G1 to S phase transformation in GC cells. We also demonstrated that APY0201 increased expression levels of p21 in GC transplanted tumor tissues. These results suggest that APY0201 blocks G1 phase to S phase transition by inhibiting the activity of cyclin-CDK complexes in GC cells.

In summary, this study showed that APY0201 has a significant inhibitory effect on GC cell proliferation. APY0201 blocked the autophagic flux of GC cells by damaging the lysosomal degradation function, causing accumulation of autophagosomes, thereby inhibiting the proliferation of GC cells. In addition, APY0201 induces G1/S phase arrest of GC cells. Therefore, APY0201 could be a potential therapeutic agent for GC.

## Data Availability

The original data are available upon reasonable request by writing to the corresponding author.
